# Charge-Transfer Controlled Crystallization of a Model Oligomer for Donor-Acceptor-Polythiophenes

**DOI:** 10.3390/ma3031904

**Published:** 2010-03-17

**Authors:** Ralph Rieger, Volker Enkelmann, Klaus Müllen

**Affiliations:** Max Planck Institute for Polymer Research, Ackermannweg 10, 55128 Mainz, Germany; E-Mails: rieger@mpip-mainz.mpg.de (R.R.); ve@mpip-mainz.mpg.de (V.E.)

**Keywords:** conjugated oligomer, donor-acceptor, charge-transfer, crystal engineering, molecular packing, energy levels

## Abstract

A model donor-acceptor oligomer consisting of benzodithiophene-diketone and thiophene has been investigated with regard to its molecular packing and opto-electronic properties. The crystal structure suggests that the packing is dominated by charge-transfer interactions between the electron-rich part of the molecule and the electron-poor part. A series of observations corroborate this assumption, among them are a charge-transfer band in the film absorption spectra and exceptionally low π-π distances. A detailed analysis of the energetic levels of the present system reveals that only the HOMO level of the acceptor is shifted by conjugation to the donor. The results can contribute to the development of improved materials for organic electronics.

## 1. Introduction

Conjugated donor-acceptor molecules play an important role in almost every field of materials research [1,2,3]. In recent years, much research has been dedicated to the development of organic materials for electronic applications [4,5]. Conjugated polymers incorporating donor and acceptor subunits are among the most promising materials showing high performances in field-effect transistors or solar cells [6]. However, the relevant driving forces for the molecular packing are unclear so far. To further improve the performance of the materials in devices, a better understanding of the intra- and intermolecular interactions of donor-acceptor molecules is of special importance. As polymers are very hard to study in detail, defined small molecules and oligomers serve as model systems for accurate investigations [7].

In this work, benzo[2,1-b;3,4-b’]dithiophene-5,6-dione (**1**) is chosen as model system, because it resembles the repeat unit of a polythiophene similar to successful polymers [8,9,10]. Furthermore, its quinoid structure represents a strong acceptor moiety that can be converted into many other interesting functionalities such as benzothiadiazole [11], a very useful building block for organic electronic materials. In combination with additional thiophene units, a donor-acceptor oligomer is available, which is structurally similar to many polymers of interest.

## 2. Results and Discussion 

**Figure 1:**
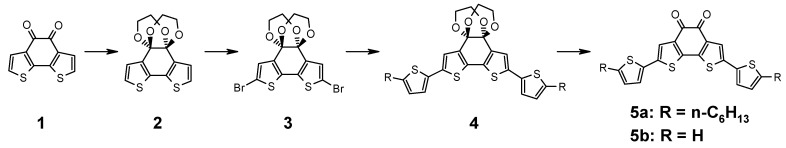
Synthesis of donor substituted benzo[2,1-b;3,4-b’]dithiophene-5,6-dione.

The synthesis is outlined in Figure 1. Benzodithiophene-dione (**1**) is functionalized by a Stille coupling reaction. As the α-diketone does not tolerate the required reaction conditions, it needs to be protected. For this reason, benzodithiophene-dione (**1**) is reacted with ethylene glycol catalyzed by trimethylchlorosilane as described in the literature to afford the bisdioxane derivative **2** [12]. After NBS bromination, compound **3** is obtained which is subject to a Stille coupling with 2-hexyl-5-tributylstannyl-thiophene to afford the protected precursor **4**. The introduction of alkyl chains makes this model system more similar to the polymers where alkyl chains are needed for solubility. The protection group is removed by acid hydrolysis using aqueous tetrafluoroboronic acid to obtain the deep blue substance **5a**. Remarkably, the reaction only proceeds as expected when using a 2:1 (v:v) mixture of dichloromethane and THF as solvent, the pristine solvents leave **4** unaffected. Both reactant and product are well soluble in both solvents.

**Figure 2:**
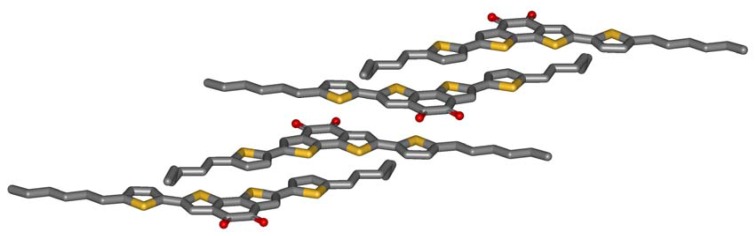
Crystal structure of **5a**.

Single crystals of **5a** are grown by slow evaporation of a dichloromethane solution. Figure 2 shows the crystal structure obtained by X-ray analysis. The striking feature of the molecular packing is the sandwiching of the electron deficient benzodithiophene-dione by two electron-rich thiophenes of adjacent molecules. The centroid-centroid distance between the donor and the acceptor of the next molecule (3.7 Å) only slightly exceeds the interlayer distance formed by the aromatic planes of the molecules (3.5 Å). The driving force for this arrangement is obviously an intermolecular charge-transfer interaction. Three observations further support this thesis: Firstly, microphase separation does not drive the aromatic and the aliphatic parts of the molecule apart. The molecules allow contacts of aromatics and alkyl chains of adjacent molecules. Secondly, half of the hexyl chains are in a gauche conformation which is needed to enable this stacking. Typically, alkyl chains prefer all-trans conformations in crystals, so an energy price is paid by the molecules to form intermolecular donor-acceptor contacts. Thirdly, the molecules do not show close sulfur-sulfur distances which other thiophene containing molecules do. All these unfavorable constellations are overcompensated by the intermolecular charge-transfer interaction.

**Figure 3:**
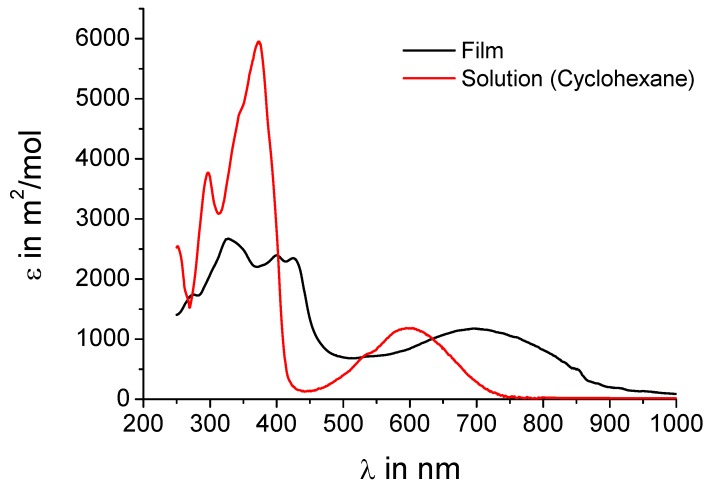
UV-vis absorption spectra of **5a** in solution (cyclohexane, 10^-5^ M) and as film.

UV-vis absorption spectra shall further corroborate the assumption that intermolecular charge-transfer interactions are present in the crystal. Figure 3 shows both the spectrum of **5a** from solution and in the film. The spectra of **5b** are almost identical as expected when just replacing alkyl chains by hydrogen and are thus not included in the figure. The concentration of the solution (10^-5^ M) should be low enough that no intermolecular charge-transfer interactions influence the absorption behavior. In solution a peak at 600 nm is observed indicating an effective intramolecular donor-acceptor interaction. In the film, where the donors sandwich the acceptors, an intense peak at 700 nm with an onset of even 900 nm is present, obviously a charge-transfer band. This strongly supports the hypothesis of an intermolecular charge-transfer complex in the solid.

**Figure 4:**
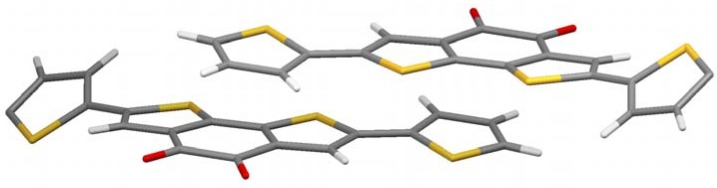
Crystal structure of **5a**.

To investigate the influence of the alkyl chains, the unsubstituted parent molecule (**5b**) was synthesized by reacting molecule **3** with 2-tributylstannylthiophene and subsequent removal of the ethylene glycol protection groups (Figure 1). Single crystals suitable for X-ray analysis are grown by slow evaporation of a THF solution. Figure 4 shows the crystal structure. It is a bit more complex, but a clear preference for the thiophenes to come in close contact to the acceptor part is obvious again. A pairwise donor-acceptor interaction can be observed in which the electron rich thiophene unit of the one molecule stacks on top of the electron-deficient diketone unit of the other molecule and vice versa, similar to the alkylated case described above. The distance between donor and acceptor planes in this case is very low (3.2 Å) considering the space demand of the large sulfur atoms. With hexyl chains the corresponding distance is 3.5 Å, but still closer than in conjugated donor-acceptor polymers (more than 3.7 Å) [13,14]. It can be concluded that the charge-transfer effect brings the molecule into very close contact, if no steric strain, e.g., by alkyl chains, is built up. 

Another interesting feature in crystal structure **5a** is the twist of the thiophene on the right and left of Figure 4. They are rotated out plane formed by the π-system of the other part of the molecule by 30.4°. Those thiophenes which stand in close proximity to the acceptor of the adjacent molecule, however, are coplanar to the acceptor they are bond to. This is another indication that the charge-transfer dominates the crystal structure, as the present conformation reduces the resonance energy of the molecule in contrast to a fully planar conformation. The charge-transfer seems to overcompensate this unfavorable energetic situation.

**Figure 5:**
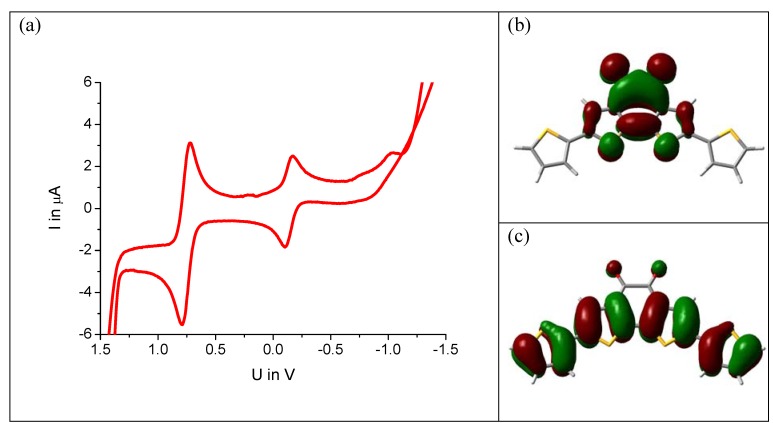
(a) Cyclovoltagram of compound **5a** (1 mM in DMF, ferrocene standard, voltage against NHE). (b) Electron density surface of the LUMO and (c) HOMO, calculated by DFT (B3LYP, 6–31G).

Knowledge of intramolecular donor-acceptor interactions is very important for energy alignments to electrode materials and band gap tuning for maximum absorption in solar cells [17,18,19,20]. The model system (**5**) is investigated by cyclovoltammetry. The molecule is dissolved in anhydrous DMF containing tetrabutylammonium perchlorate as conductive salt and ferrocene as internal standard. The cyclovoltagram of Figure 4a shows a reduction peak at −0.2 V against NHE. Surprisingly, exactly the same value is found for the unsubstituted benzodithiophene-dione (**1**) under the same measurement conditions. A DFT calculation for this molecule reveals that, in fact, the LUMO energy level remains unchanged by the substitution. The electron density surface is depicted in Figure 5b showing no electron density on the thiophenes but only on the benzodithiophene-dione unit.

The HOMO, in contrast, is strongly affected by substitution. As Figure 4c shows, the π-electrons of the thiophene units participate significantly. This is in line with the absorption spectrum of the compound, which undergoes a strong red shift upon substitution, already visible by eye: **1** is a red, **5a **and** 5b** are blue compounds. Thus, only the upward shift of the HOMO level reduces the optical gap. This means that systems showing ambipolar transport are readily accessible by this design concept: the LUMO level remains as low as in the acceptor alone facilitating electron injection. The HOMO can be pushed up by the donor as needed for efficient hole injection.

## 3. Experimental Section

All chemicals were used as received unless otherwise stated.

*5,5’-Dibromobenzo[2,1-b;3,4-b']dithiophene-4,5-ethyleneoxolane* (**3**): 154 mg (0.5 mmol) benzo[2,1-b;3,4-b']dithiophene-4,5-ethyleneoxolane [12] are dissolved in 5 ml DMF. 178 mg (1 mmol) *N*-bromosuccinimid are added. The mixture is stirred at room temperature for two hours, diluted with diethylether, extracted three times with water, and dried. After evaporation of the solvent, the residue is chromatographed on silica gel with dichloromethane as eluent. The obtained solid is recystallized from ethyl acetate to afford 130 mg (56%) of slightly yellow needles. Mp = 130 °C; MS (FD, 8 kV) m/z 465.9 g/mol – calculated: 465.8 g/mol for C_14_H_10_Br_2_O_4_S_2_^1^H-NMR (250 MHz, CD_2_Cl_2_, RT, δ in ppm) 7.14 (s, 2H), 4.1 (m, 4H), 3.6 (m, 4H); ^13^C-NMR (62.5 MHz, CD_2_Cl_2_, RT, δ in ppm) 137.1, 133.9, 129.0, 111.8, 93.4, 67.2, 62.1; elemental analysis: found 35.76% C, 1.83% H, 13.45% S–calculated: 36.07% C, 2.16% H, 13.76% S.

*5,5’-(5-hexylthiophene-2-yl-)benzo[2,1-b;3,4-b']dithiophene-4,5-ethyleneoxolane* (**4a**): 92 mg (0.2 mmol) 5,5’-Dibromobenzo[2,1-b;3,4-b']dithiophene-4,5-ehyleneoxolane and 228 mg (0.5 mmol) 5-hexyl-2-tributylstannylsthiophene are dissolved in 5 ml anhydrous DMF and degassed. 10 mg (1 µmol) tetrakis(triphenylphsophine)-palladium is added. The resulting mixture is stirred for two hours under an argon atmosphere. After cooling, the solvent is evaporated in high vacuum. The residue is chromatographed on silica gel with dichloromethane as eluent. 110 mg of a yellow substance are obtained (86%). Mp = 148 °C; MS (FD, 8 kV) m/z 640.5 g/mol – calculated: 640.2 g/mol for C_34_H_40_O_4_S_4_^1^H-NMR (300 MHz, CD_2_Cl_2_, RT, δ in ppm) 7.16 (s, 2H), 7.02 (d, *J* = 3.6 Hz, 2H), 6.72 (d, *J* = 3.6 Hz, 2H), 4.2 (m, 4H), 3.7 (m, 4H), 2.81 (t, *J* = 7.5 Hz, 4H), 1.69 (quin, *J* = 7.1 Hz, 4H), 1.4–1.3 (m, 12H), 0.93 (t, *J* = 6.5 Hz); ^13^C-NMR (75 MHz, CD_2_Cl_2_, RT, δ in ppm) 146.9, 137.4, 137.0, 134.6, 131.2, 125.6, 124.3, 121.7, 93.9, 62.2, 32.2, 32.1, 30.7, 29.3, 23.2, 14.4; elemental analysis: found 63.40% C, 6.03% H, 19.64% S – calculated: 63.71% C, 6.29% H, 20.01% S.

*5,5’-Di(5-hexylthiophene-2-yl-)benzo[2,1-b;3,4-b']dithiophene-4,5-diketone* (**5a**): 64 mg (0.1 mmol) 5,5’-(5-hexylthiophene-2-yl-)benzo[2,1-b;3,4-b']dithiophene-4,5-ehyleneoxolane are dissolved in 5 ml dichloromethane/THF (2:1) and degassed. 2 ml tetrafluoroboronic acid (50% in water) are added. The mixture is intensively stirred for two days, extracted with dichloromethane and chromatographed on silica gel with dichloromethane as eluent. 45 mg of a blue solid are obtained (82%). Mp = 150 °C; MS (FD, 8 kV) m/z 552.2 g/mol – calculated: 552.1 g/mol for C_30_H_32_O_2_S_4_; ^1^H-NMR (250 MHz, CD_2_Cl_2_, RT, δ in ppm) 7.30 (s, 2H), 7.03 (d, *J* = 3.6 Hz, 2H), 6.71 (d, *J* = 3.6 Hz, 2H), 2.79 (t, *J* = 7.5 Hz, 4H), 1.67 (quin, *J* = 7.0 Hz, 4H), 1.4–1.2 (m, 12H), 0.90 (t, *J* = 6.6 Hz); ^13^C-NMR (62.5 MHz, CD_2_Cl_2_, RT, δ in ppm) 174.6, 148.6, 141.5, 139.0, 136.2, 132.8, 125.8, 125.7, 122.1, 32.1, 32.0, 30.7, 29.3, 23.2, 14.4; elemental analysis: found 64.90% C, 5.76% H, 23.22% S – calculated: 65.18% C, 5.83% H, 23.20% S.

*5,5’-Dithien-2-ylbenzo[2,1-b;3,4-b']dithiophene-4,5-ethyleneoxolane* (**4b**): 47 mg (0.1 mmol) 5,5’-dibromobenzo[2,1-b;3,4-b']dithiophene-4,5-ehyleneoxolane are dissolved in 2 ml anhydrous DMF under argon. 93 mg (0.25 mmol) 2-tributylstannylthiophene are added, followed by 5 mg (5 µmol) Pd(PPh_3_)_4_. The mixture is heated for two hours to 100 °C, diluted with diethylether, extracted with water three times, dried. The solvents are evaporated, the residue is purified by preparative chromatography on silica gel with a gradient of petroleum ether – dichloromethane (9:11 v/v) to pure dichloromethane. After crystallization from ethyl acetate at −20 °C, 28 mg of a yellow crystalline substance are obtained (59%). Mp. = 262 °C, MS (FD, 8 kV) m/z 471.8 g/mol – calculated: 472.0 g/mol for C_22_H_16_O_4_S_4_; ^1^H-NMR (250 MHz, CD_2_Cl_2_, RT, δ in ppm) 7.28 (dd, *J*_1_ = 5.1 Hz, *J*_2_ = 1.1 Hz, 2H), 7.26 (s, 2H), 7.23 (dd, *J*_1_ = 3.6 Hz, *J*_2_ = 1.1 Hz, 2H), 7.05 (dd, *J*_1_ = 5.1 Hz, *J*_2_ = 3.6 Hz, 2H), 4.15 (m, 4H), 3.70 (m, 4H); ^13^C-NMR (62.5 MHz, CD_2_Cl_2_, RT, δ in ppm) 137.7, 137.2, 136.6, 131.7, 128.6, 125.6, 124.6, 122.5, 93.8, 62.2; elemental analysis: found 56.01% C, 3.38% H, 26.78% S – calculated: 55.91% C, 3.41% H, 27.14% S.

*5,5’-Dithiophene-2-yl-benzo[2,1-b;3,4-b']dithiophene-4,5-diketone* (**5b**): 150 mg (0.32 mmol) 5,5’-thiophene-2-yl-benzo[2,1-b;3,4-b']dithiophene-4,5-ehyleneoxolane are dissolved in a mixture of 5 ml dichloromethane and 2.5 THF. After degassing, 3 ml tetrafluoroboronic acid (50% in water) are added. The mixture is intensively stirred overnight, extracted with dichloromethane and chromatographed on silica gel with dichloromethane as eluent. 100 mg of a blue solid are obtained (81%). Mp = 292 °C; MS (FD, 8 kV) m/z 383.7 g/mol – calculated: 383.9 g/mol for C_18_H_8_O_2_S_4_; ^1^H-NMR (300 MHz, THF-d_8_, RT, δ in ppm) 7.57 (s, 2H), 7.47 (dd, *J*_1_ = 5.1 Hz, *J*_2_ = 1.1 Hz, 2H), 7.40 (dd, *J*_1_ = 3.6 Hz, *J*_2_ = 1.1 Hz, 2H), 7.09 (dd, J_1_ = 5.1 Hz, J_2_ = 3.6 Hz, 2H); ^13^C-NMR (175 MHz, THF-d_8_, RT, δ in ppm) 172.3, 138.9, 136.5, 136.1, 134.3, 127.2, 125.5, 124.5, 121.5; elemental analysis: found 56.46% C, 2.31% H, 33.08% S – calculated: 56.23% C, 2.10% H, 33.35% S.

Solution UV-vis spectra were recorded at room temperature on a Perkin-ElmerLambda 100 spectrophotometer. Solvents of spectroscopic grade were employed. The baseline was corrected by substracting a measurement of the cuvette filled with pure solvent used for the measurement.

Cyclic volatammetry was measured on a Princeton Applied Research Parstat 2273 instrument with anhydrous solvents under argon atmosphere. Tetrabutylammoniumperchlorate was used as conductive salt at a concentration of 0.1 mol/L. Ferrocen was added as internal standard (1 mM). A platinum working electrode (0.5 mm diameter), a platinum wire as counter electrode, and a silver wire as quasi-reference electrode were used. The peaks were calibrated according to the oxidation peak of ferrocene. Half-step potentials were used for the evaluation.

The single crystal analysis was performed on a Nonius-KCCD diffractometer with a Mo-K_α_ (λ = 0.71923 Å, graphite monochromatized) at a temperature of 150 K. The structures were solved by direct methods (Shelxs) and refined on F with anisotropic temperature factors for all non-hydrogen atoms. The H atoms were refined with fixed isotropic temperature factors in the riding mode.

Structure **5a**: C_30_H_32_O_2_S_4_, M_r_ = 552.85 g/mol, triclinic, space group P 1 2_1_/n, a = 10.7680(4) Å, b = 11.3767(4) Å, c = 12.2773(3) Å, α = 100.264(2)*°*, β = 105.346(2)*°*, γ = 103.265(2)*°*, V = 1365.21(8) Å^3^, Z = 2, ρ_calcd_ = 1.345 g/cm^3^, μ = 0.375, 2θ_max _= 29.569*°*, 19251 reflections measured, 7575 unique, 5403 observed, R_int_ = 0.066, R = 0.0394, R_w_ = 0.0478, Cambridge Crystallographic Data Centre identifyer: CCDC-740343.

Structure **5b**: C_18_H_7_O_2_S_4_, M_r_ = 383.52 g/mol, monoclinic, space group P 1 2_1_/n, a = 15.9180(6) Å, b = 5.47300(10) Å, c = 17.6830(6) Å, β = 96.4220(14)*°*, V = 1530.86(8) Å^3^, Z = 4, ρ_calcd_ = 1.664 g/cm^3^, μ = 0.628, 2θ_max_ = 29.523*°*, 14386 reflections measured, 4276 unique, 3114 observed, R_int_ = 0.080, R = 0.0502, R_w_ =0.0572, Cambridge Crystallographic Data Centre identifyer: CCDC-740344.

## 4. Conclusions

In summary, it could be shown that a model system for donor-acceptor polythiophenes crystallizes with the donor part on top of the acceptor driven by an intermolecular charge-transfer. A series of results support this thesis: (i) energetically unfavorable molecular packing to establish donor-acceptor contact in the crystal, (ii) a charge-transfer band in the film UV-vis absorption spectrum, (iii) extremely low π-π distances in the absence of alkyl substituents. The intramolecular donor-acceptor interaction efficiently lowers the optical gap, but solely by shifting the HOMO level upwards, the LUMO is unaffected. 

These observations have highly important consequences for the design of new donor-acceptor polymers: the intermolecular charge-transfer dominated crystallization reduces the interchain distances. On the one hand, this is very useful to obtain high charge-carrier mobilities as hopping distances and thus potential barriers decrease [15,16]. On the other hand, strong interchain interactions and low distances reduce the solubility in the corresponding polymer. The steric demand of alkyl chains increases the distance giving rise to better solubility, but lowering the charge-carrier mobility. The position of the alkyl chain, furthermore, strongly affects the interchain distances. An optimum compromise has to be found to not increase too much the distance but still obtain enough solubility for processing. The experimental results presented here contribute to a better understanding of donor-acceptor polymers and can guide the design for new materials with improved device performances.
